# Direct synthesis of lemongrass *(Cymbopogon citratus L.)* essential oil*-*silver nanoparticles (EO-AgNPs) as biopesticides and application for lichen inhibition on stones

**DOI:** 10.1016/j.heliyon.2022.e09701

**Published:** 2022-06-10

**Authors:** Riyanto, Meike Mulwandari, Luthfiah Asysyafiiyah, Melisa I. Sirajuddin, Nahar Cahyandaru

**Affiliations:** a Department of Chemistry, Islamic University of Indonesia, Jalan Kaliurang KM 14,5 Sleman, Yogyakarta, 55584, Indonesia; b Borobudur Conservation Office, Borobudur, Magelang, Central Java, Indonesia

**Keywords:** Essential oils, Silver nanoparticle, Lemongrass, Biopesticides, Lichens

## Abstract

Lemongrass essential oil *(Cymbopogon citratus L.)* is used directly to kill lichens and has many disadvantages such as being less effective, volatile, and inefficient. Lichens are a type of microbe that grows in rocks and cause biodeteriorations of rock material because they are highly erosive. Therefore, this research aims to investigate the direct synthesis of lemongrass *(Cymbopogon citratus L.)* essential oil-silver nanoparticles (EO-AgNPs) as biopesticides and application for lichens inhibition on stones. This was carried out in order to improve the performance and effectiveness of biopesticides which is excellent in killing lichens on stone surfaces. However, it has several disadvantages, such as not being economical, slow performance, and high volatility. The EO-AgNPs nanoparticles were produced by adding AgNO_3_ powder directly to lemongrass essential oil. They were then observed to know the effect of variations in storage time on material stability and AgNO_3_ concentration. The synthesized material was characterized by UV-Vis Spectrophotometer, FTIR, particle size analyzer (PSA), and SEM-EDX before being tested for its effectiveness in killing lichens directly on stones and inhibition activity. The results showed that the EO-AgNPs had been successfully synthesized as indicated by the color of the clear dark brown solution in the wavelength range of 430 nm. Furthermore, after it was analyzed using PSA and SEM-EDX, EO-AgNPs had a particle size of 332 nm and were spherical with Ag, C, O content of 27.28, 57.98, and 14.74%, respectively. The antifungal activity for killing lichens based on the diameter of inhibition zone (DIZ) using EO and EO-AgNO_3_ was 14.7 mm and 20.3 mm, respectively. This shows that EO-AgNPs nanoparticles are capable of killing lichens on rock surfaces and also have a better inhibition activity than EO.

## 1 Introduction

Essential oil (EO) is a natural alternative because it has the ability to protect archaeological objects [1, 2, 3, 4, 5, 6]. It can be used to inhibit microbial growth because of its biodegradability, cost-effectiveness, environmental suitability, non-toxicity, antimicrobial, and antioxidant properties. EO is widely used as antimicrobial, anti-inflammatory, analgesic, biopesticide, antibacterial, antioxidant, antifungal, and mosquito control [7, 8, 9, 10, 11, 12]. Citronella oil is one of its commonly used component because it contains active compounds that have enormous potential as antifungals such as linalool, α-pinene, β-pinene, and menthone. Citronella and linalool belong to the group of terpenoids and monoterpenes which can suppress the growth of pathogenic fungi. These compounds are capable of inhibiting fungal metabolic processes and also interfere with their growth [11, 13, 14].

Despite their promising properties, EO-based fungicides have several disadvantages such as inefficiency, expensive, and ineffective due to oil volatilities, poor water solubility, and environmental degradation. These is associated with their chemical composition which is capable of disrupting their application. Metal nanoparticle EO is an alternative method that is used to reduce the problem of increasing effectiveness and induction of systemic activity because of its small size. These metal nanoparticles have a unique optoelectrical properties due to their localized surface plasmon resonance characteristics and a broad absorption band of the electromagnetic spectrum [15, 16]. Further research is also needed on the manufacture of biopesticide formulations based on citronella EO with silver metal (Ag) nanoparticle scale as a material for conservation of cultural heritage objects. Silver exhibits higher toxicity to various microorganisms because its attachment to the cell membrane surface significantly impairs proper functions such as respiration and permeability. Several research have used Ag metal as a precursor in the manufacture of nanoparticles [13, 17, 18, 19, 20, 21, 22, 23, 24, 25, 26, 27, 28, 29, 30, 31, 32]. In addition, the gold metal can also be used as a material for making nanoparticles [33, 34].

Nanoparticle-scale essential oils are more effective and efficient in killing fungi or lichens. Furthermore, biopesticide nanoparticles prevent the volatility of citronella essential oil, use efficiency, and leave no marks on cultural heritage objects. They have been extensively studied for various technological applications and in materials science, chemistry, physics, biology, and environmental science research [10, 35]. Nanoparticles are particles ranging in various sizes from 1 to 100 nm and most methods suggest a particle diameter of between 200 to 40 nm. These materials are used to overcome the solubility of an active substance that is difficult to dissolve, improve poor bioavailability and the absorption of a macromolecular compound, increase the stability of the active substance from environmental degradation such as enzymatic decomposition, oxidation, hydrolysis, and reduce the irritating effect of the active substance on the gastrointestinal tract [36].

Materials or structures with small sizes have different separate properties from one another. The specific characteristics of these nanoparticles depend on their size, distribution, morphology, and phase. The shape and size of Ag nanoparticles greatly determine their optical, electrical, magnetic, catalytic, and antibacterial properties. Therefore, the smaller the particle size, the greater the antimicrobial effect. Factors that affect the particle size in the synthesis are solution temperature, metal concentration, reducing agent, and reaction time. Some advantages of nanoparticles is that they are capable of penetrating intercellular spaces along with colloidal particles. Additionally, they are flexible to be combined with various other technologies. This capability opens the wide potential to be developed for various purposes and targets. Another advantage is the increased affinity of the system due to an increase in the contact surface area by the same amount [37].

The synthesis of nanoparticle can take place in liquid, solid, and gas phases, while the process of manufacturing can occur chemically or physically. The physical synthesis involves the breakdown of large materials into very smaller materials sizes (nanometer size) without changing their properties. However, that of chemical synthesis involves chemical reactions that occur from several starting materials. The formation of nanoparticles with high regularity can produce a more uniform pattern and uniform size [38, 39]. An example of the materials synthesized is Ag. Colloidal Ag has long been known to have antimicrobial properties which is influenced by several factors, such as the concentration, shape, and size of their nanoparticles, type of bacteria, number of bacterial colonies, and contact time of Ag nanoparticles with bacteria [40].

Nanoparticles can be synthesized by three methods namely chemically such as reduction [41], electrochemically and bio-reduction also called green synthesis. Reduction by biological methods or known as bio-reduction is reduction using chemicals from nature such as plant extracts and essential oils. This method is widely used compared to other methods because it has many advantages such as low cost, being environmentally friendly, and safer [42, 43]. Silver nanoparticles have been made by several different methods and conditions such as via chemical reduction, photochemistry, sonochemistry, ultrasonic radiation, and solvothermal synthesis. The chemical reduction is an effective method that is used to produce Ag nanoparticles due to the fact that it is easy to perform, fast, cheap, and use low temperatures. Furthermore, lemongrass EO can be used for the application of metal nanoparticle such as the synthesis of Ag nanoparticles from Cymbopogon leaf extract as an antibacterial [44].

Metal nanoparticles have very useful properties in various fields such as having high surface area, catalytic properties, and antibacterial ability. Silver metal nanoparticles have very promising properties like antibacterial, antifungal, and antiviral materials. Silver metal nanoparticles are synthesized by various methods such as reduction by radiation, electrochemistry, reduction with natural (biological) materials, and reduction with various solvents. These various methods aim to reduce Ag^+^ ions to Ag^o^. The method that is currently being developed is the reduction method with natural materials. The biological reduction method has the advantages of being environmentally friendly, non-toxic, and lower in cost [45].

Lichens are plants that are formed from a combination of fungi and algae. The symbiosis between fungi and algae is two separate organisms. They are very easy to grow on rotting wood surfaces, roofs of houses, and various rocks. Lichens can grow and develop from organic and inorganic materials contained in rocks. Stone overgrown by lichens will be porous and damaged. They will destroy cultural heritage objects made of stone, wood, and cement. Lichens are plants that have biodeteriogenic properties and are the most aggressive that can damage cultural heritage objects. Cultural heritage objects will suffer more severe damage if they are outside the room [46]. This research presents the results on the synthesis of EO-AgNPs using citronella EO which was obtained via steam distillation and AgNO_3_ powder. The EO-AgNPs were synthesized directly because water as a solvent is not soluble in EO. Therefore, AgNO_3_ powder was added directly to EO in order to save synthesis costs.

## 2 Experimental

### 2.1 Synthesis of lemongrass essential oil (EO)

Lemongrass EO is obtained from the steam distillation process. The distillation apparatus is made of stainless steel with a capacity of 10 kg. Citronella leaf samples ready for harvest were cut and chopped before being placed into the distillation kettle which lasted for 6 h. The EO was collected in a separator and separated from the water by a separating funnel and was purified by adding anhydrous sodium sulfate. Lemongrass EO was analyzed for chemical compounds using Gas Chromatography-Mass Spectrometry (GC-MS) from Shimadzu QP2010 SE.

### 2.2 Synthesis essential oil-silver nanoparticle (EO-AgNPs)

**Figure 1:**
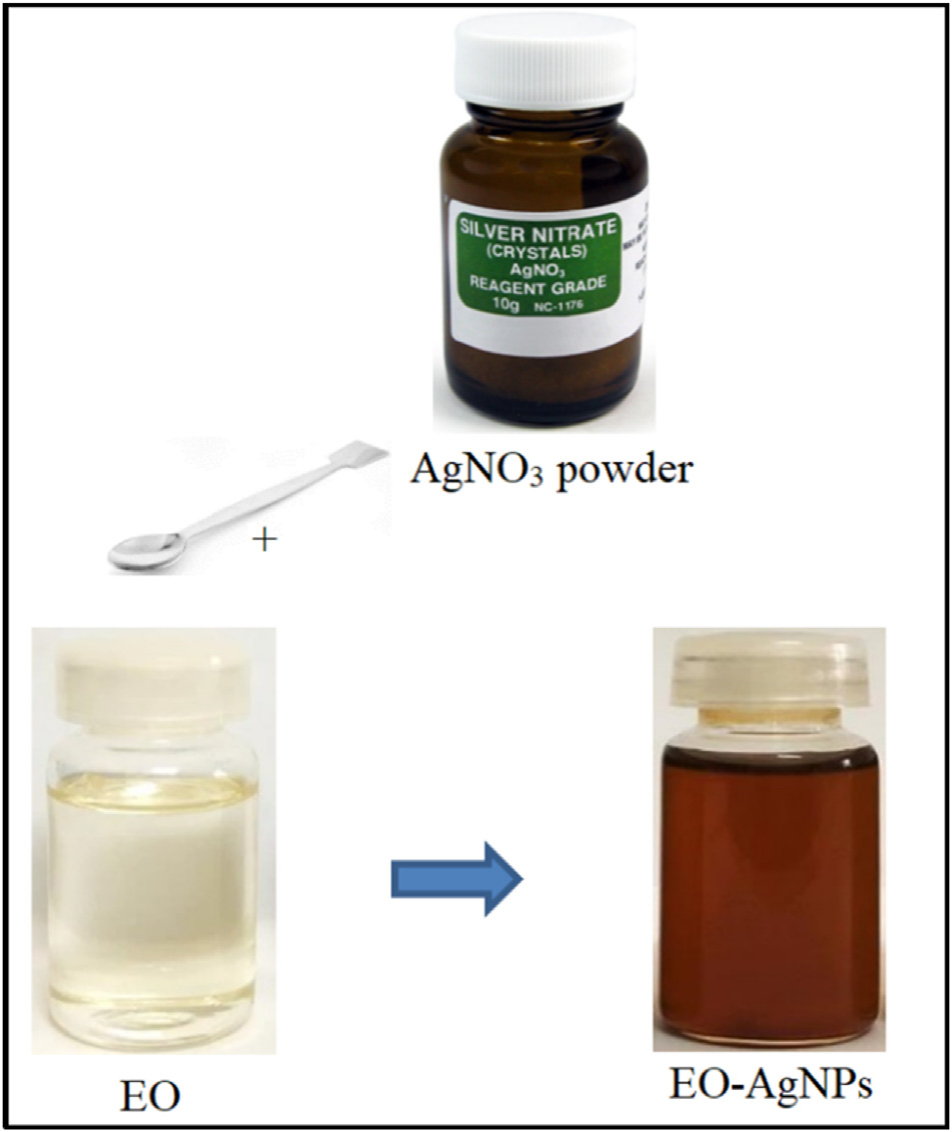
The scheme of the direct synthesis of EO-AgNPs.

Synthesis of EO-AgNPs was carried out by mixing AgNO_3_ powder directly in citronella EO in a 100 mL beaker. The mixture was stirred until homogeneous and stored in the dark for 24 h at room temperature. The stability of the EO-AgNPs formed was observed for 1, 3, 5, 7, 9, and 11 days with a UV-Vis spectrophotometer. A study of variations was also carried out on the concentration of silver nitrate (AgNO_3_) of 2, 4, 6, 8,10 mM on EO-AgNPs material. The working scheme of the synthesis of EO-AgNPs is shown in Figure 1.

### 2.3 Characterizations of EO-AgNPs

The synthesized EO-AgNPS material was characterized using a UV-Vis Spectrophotometer (Hitachi UH 5300), Fourier-Transform Infrared Spectroscopy (FTIR) (Perkin Elmer Spectrum Version 10.5.1), Particle Size Analyzer (PSA) (Horiba SZ-100, Japan), and Scanning Electron Microscope-Energy Dispersive X-Ray (SEM-EDX) (Phenom-World).

### 2.4 Application EO-AgNPs for inhibition of lichens

#### 2.4.1 Application of EO-AgNPs for the direct spray of lichens on the stone surface

EO-AgNPS was placed in a spray bottle and then sprayed on the lichens growing on the rocks. This was followed by visual observation with a handy microscope for a duration of 24 h. Furthermore, the effectiveness of EO-AgNPs in killing lichens was compared with control (EO) and blank (water) materials.

#### 2.4.2 Application EO-AgNPs for inhibition of lichens using inhibition factor

All equipment used for the inhibition test was washed and rinsed with 96% alcohol, then wrapped in paper and sterilized using an oven at 180 °C for 2 h. The medium used for specific fungi in this research was Czapek Dox Agar (CDA). CDA was dissolved using distillate water and heated in an Erlenmeyer covered with cotton. The pH value was checked (25 °C) 7.3 ± 0.2 before being sterilized using an autoclave for 2 h (121 °C). The sterile media was transferred to sterile petri dishes and stored for 24 h at room temperature to ensure that the media was not contaminated. Lichen samples were scraped using a scuffle and put in a tube. The samples were washed with 0.9% NaCl solution and centrifuged at 1,000 rpm for 10 min to separate the lichen and impurities. The ose wire was burned first, then the lichen sample was taken using the end of the ose and planted in agar media. After planting, the petri dishes were covered with plastic wrap and incubated for 5–7 days. Afterwards, the fungi were selected as lichens and separated using the shear method to separate fungal cells. The obtained fungi were inoculated (planted on new sterile media) and multiplied for stock and assays using Ag nanoparticles. The fungal media that has been obtained from the isolation process was then tested for effectiveness against inhibition by placing a small filter paper in the middle of the cup which has been dripped with EO-AgNPs, EO, and distillate water. Antifungal activity for lichens based on the diameter of inhibition zone (DIZ) of EO-AgNPs was compared against the EO and blank (distillate water).

## 3 Results

### 3.1 Characterizations of lemongrass essential oil using GC-MS

**Figure 2:**
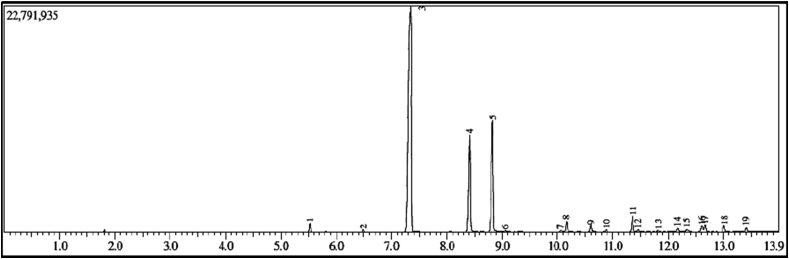
Chromatogram analysis results of EO of lemongrass leave *Cymbopogon citratus* L.

Analysis of chemical compounds in the essential oil of *Cymbopogon citratus L.* lemongrass leaves was carried out by GC-MS. MS analysis was performed by matching the fragmentation pattern of the mass spectra with the mass spectrometer database from the National Institute of Standards and Technology Mass Spectral. The refractive index at 28.3 °C of lemongrass EO was 1.4623. This indicates that the EO contains chemical compounds from the monoterpene, sesquiterpene, and their derivatives groups. The value of the specific gravity of the EO obtained was 0.875 g/cm^3^. Furthermore, the chromatogram of citronella leaf EO analysis by GC-MS is shown in Figure 2, while the chemical composition of lemongrass EO is shown in Table 1.

**Table 1:** Percentage of compounds in *Cympogon nardus* L EO identified by GC-MS.

No.	Name of compounds	Retention time (min)	Area (%)
1	1-Limonene	5.523	0.97
2	Linalool	6.492	0.28
3	Citronella	7.343	56.96
4	Beta-Citronellol	8.417	13.72
5	Geraniol	8.828	17.81
6	Citral	9.059	0.16
7	Cyclohexanol	10.062	0.27
8	Citronellal acetate	10.163	1.32
9	Nerylacetate	10.594	0.93
10	(-)- beta-Elemene	10.881	0.35
11	Trans-Caryophyllene	11.356	2.25
12	Alpha-Bergamotene	11.452	0.36
13	Alpha-Humulene	11.818	0.27
14	Germacrene D	12.173	0.51
15	1,6-Cyclodecadiene	12.339	0.49
16	Torreyol	12.611	0.96
17	Delta-Cadinene	12.662	0.89
18	Elemol	13.008	0.92
19	Germacrene D-4-ol	13.409	0.58

**Figure 3:**
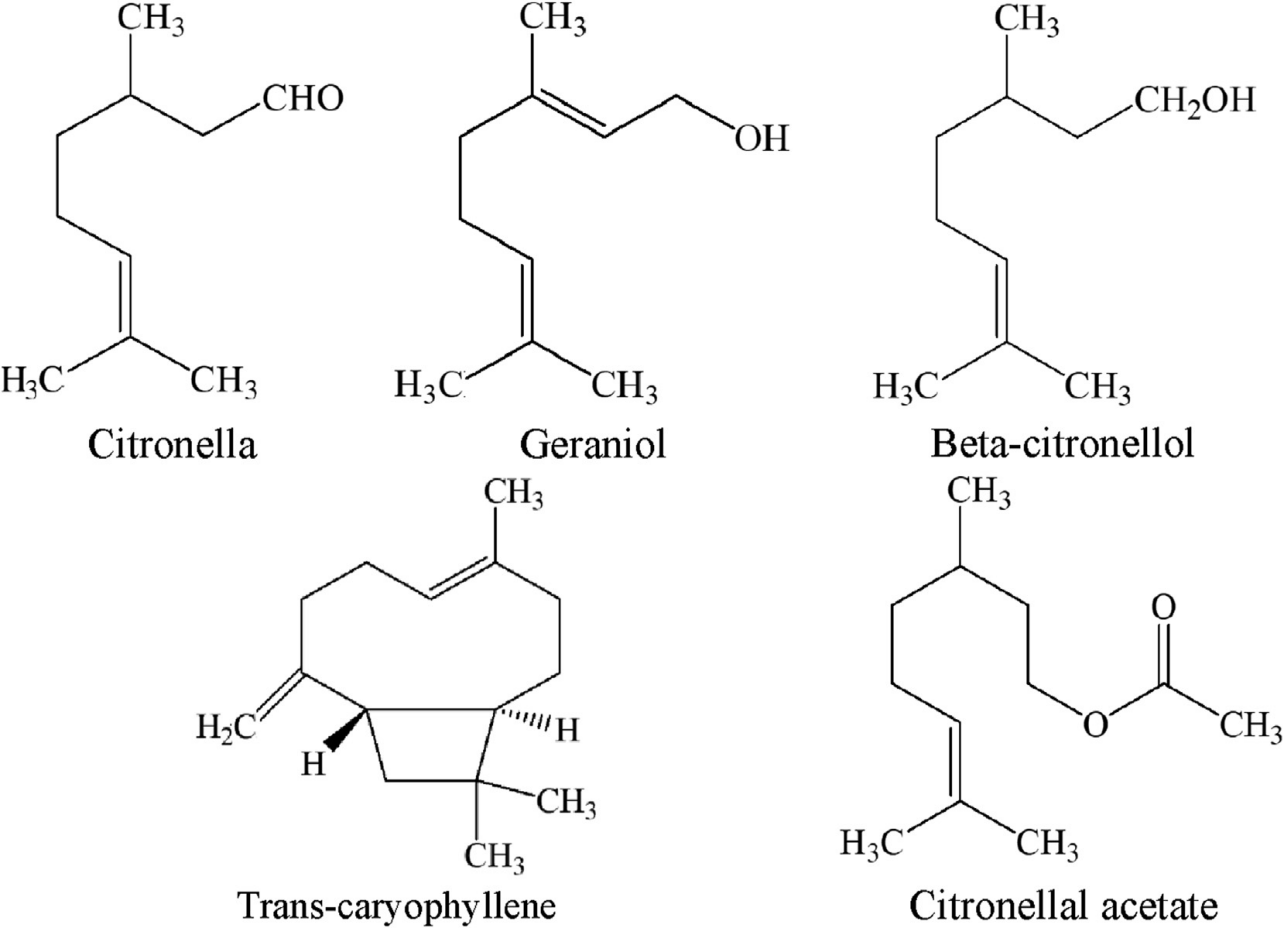
Molecular structures of the lemongrass EO *(Cymbopogon citratus L.)*.

Table 1 shows that *Cymbopogon citratus L.* EO contains chemical compounds with the main components being citronellal (56.96%), geraniol (17.81%), beta-citronellal (13.72%), trans-caryophyllene (2.25%), and citronellal acetate (1.32%). The molecular structure of a chemical compound is shown in Figure 3.

### 3.2 The effect of storage time on the stability of EO-AgNPs

**Figure 4:**
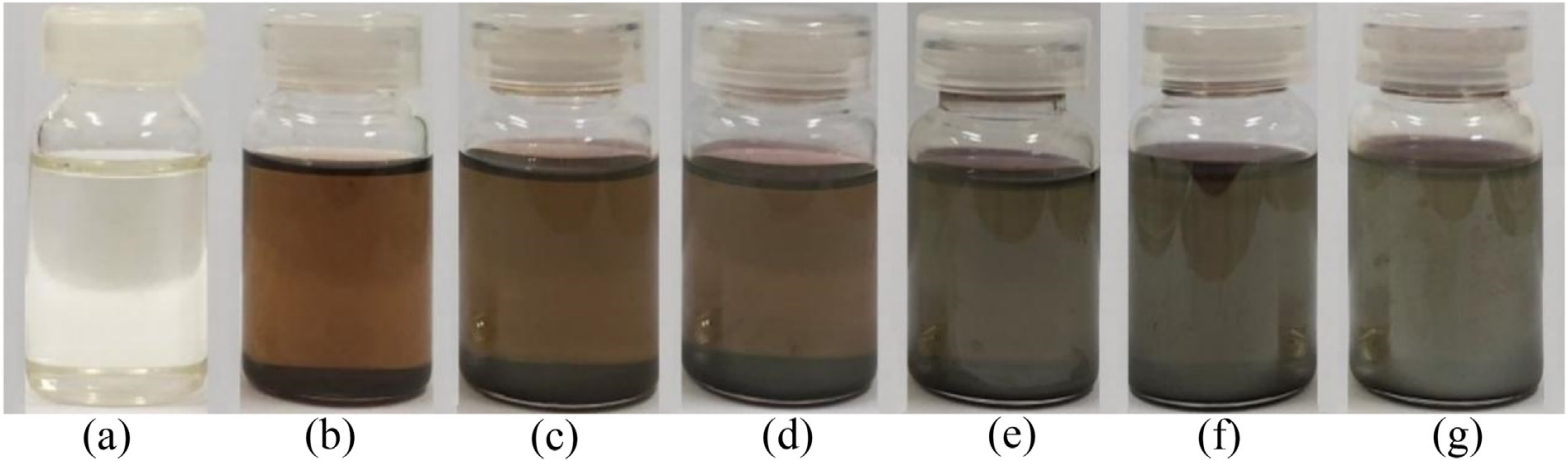
The visual observations of EO-AgNPs stored for (a) EO (b) 1 (c) 3 (d) 5 (e) 7 (f) 9 and (g) 11 days.

**Figure 5:**
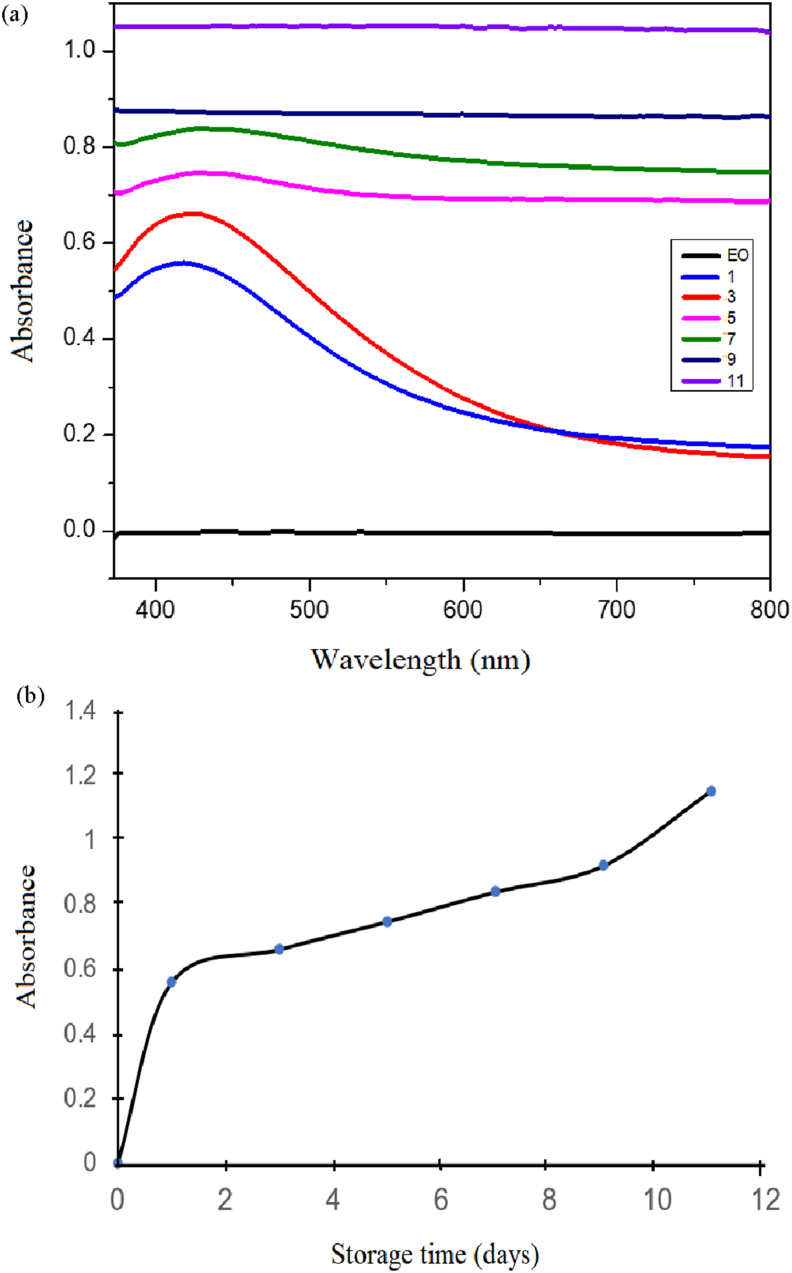
Spectra UV-Vis the effect of time (1, 3, 5, 7, 9 and 11 days) to the stability of EO-AgNPs (a) and storage time to absorbance (b).

The principle of green synthesis of EO-AgNPs was used to utilize biological materials such as plants that are used as bio-reductions, one of which is citronella *(Cymbopogon citratus L)*. Silver nanoparticles (EO-AgNPs) were synthesized from *Cymbopogon citratus L*. lemongrass EO by the bio-reduction method. Synthesis of EO-AgNPs was confirmed by visual observation of the color change of the solution because of bioreduction of Ag^+^ with EOs. The color change from pale yellow to dark brown indicates that bio-reduction of Ag+ in EO-AgNPs has taken place (Figure 4). The spectra of the analysis of EO-AgNPs using a UV-Vis spectrophotometer are shown in Figure 5.

Visually, the effect of variations in storage time on the synthesis of EO-AgNPs was shown by a brownish color change. The stability of EO-AgNPs at the variation of storage time was indicated by the maximum wavelength. EO-AgNPs had a maximum wavelength of 430 nm and absorbance in the range of 0.560–1.153 (Figures 5a and 5b). Figure 5a shows that the solution of EO-AgNPs at 9 and 11 days of storage did not form a peak at a maximum wavelength of 430 nm. This indicates that the directly synthesized EO-AgNPs were unstable, although the absorbance increased along with the storage time (Figure 5b).

The synthesized Eo-AgNPs formed at a maximum wavelength of 430 nm are characteristic of Ag nanoparticles (Ag^o^), while the wavelength of 370–400 nm represents the absorption of Ag ions (Ag^+^). The maximum wavelength of 430 nm indicates the reduction process of Ag^+^ to Ag^o^ has occurred [21, 22]. Based on the maximum wavelength obtained, the EO-AgNPs was formed from the first day (24 h). The characteristic peak of the formation of Ag nanoparticles disappeared (not formed) during storage for 9 and 11 days. The stability of the EO-AgNPs solution was known from the formation of changes in absorption peaks. Furthermore, when there is a shift in the absorption peak to a larger wavelength, it indicates that the stability of colloidal Ag nanoparticles is still low due to an agglomeration process.

### 3.3 The effect of concentration variations of AgNO_3_ to the synthesis of EO-AgNPs

**Figure 6:**
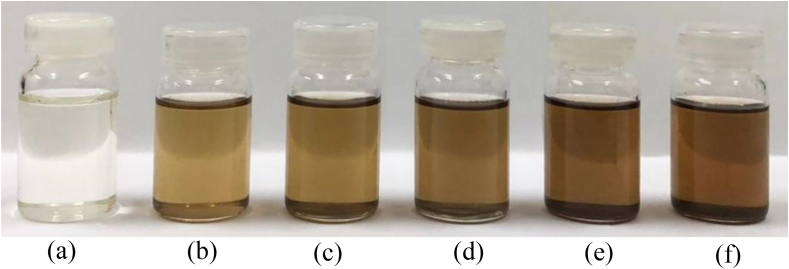
Visual EO-AgNPs using different concentration of AgNO_3_ (a) EO (b) 2 mM (c) 4 mM (d) 6 mM (e) 8 mM dan (f) 10 Mm.

**Figure 7:**
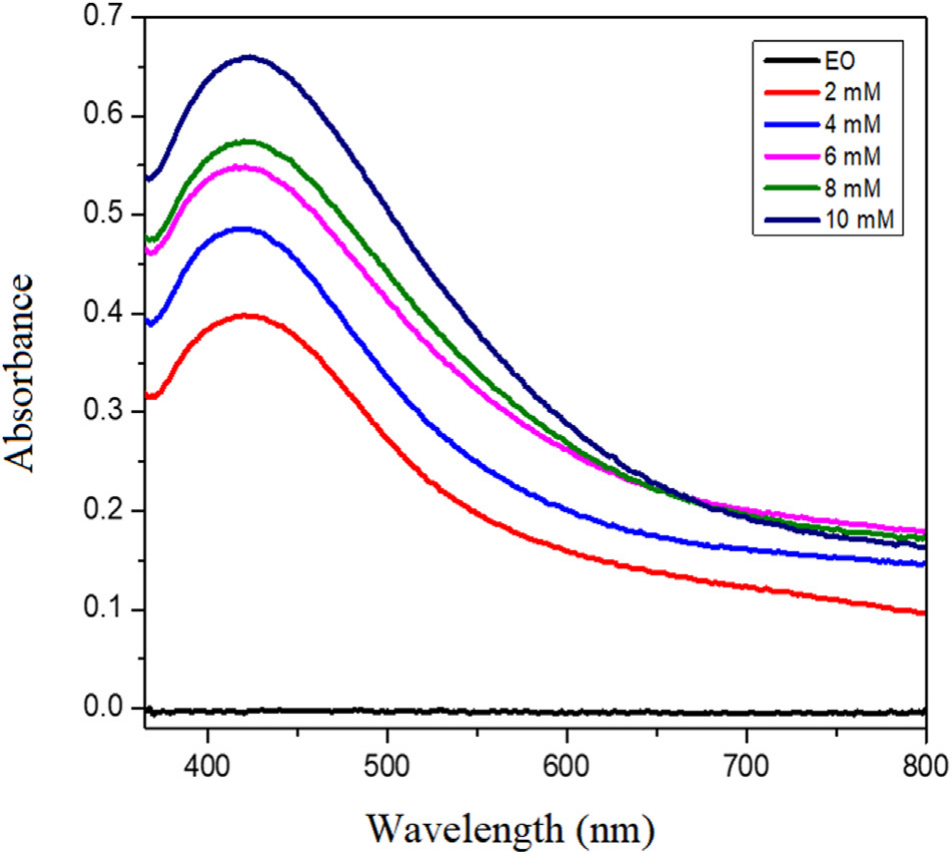
Spectra UV-Vis of the EO-AgNPs using different concentrations of AgNO_3_.

The AgNO_3_ concentration is very influential on the success of the synthesis of EO-AgNPs. The synthesis of EO-AgNPs was carried out using various concentrations of AgNO_3_, namely 2, 4, 6, 8 and 10 mM. A solution with a concentration of AgNO_3_ 2 mM has a lighter color than a solution of 10 mM Figure 6 shows a visual of the effect of AgNO_3_ concentration on the synthesis of EO-AgNPs. The higher the concentration, the darker the color formed. The results of the analysis of variations in AgNO_3_ concentration were confirmed using a UV-Vis spectrophotometer. Figure 7 shows that at concentrations of AgNO_3_ 0 mM (EO) not peak was detected at a wavelength of 440 nm. The AgNO_3_ concentrations of 2, 4, 6, 8 and 10 mM, peak was detected at a wavelength of 440 nm. The AgNO_3_ concentrations of 2, 4, 6, 8 and 10 mM resulted in a maximum wavelength of 440 nm, and absorbances of 0.392, 0.472, 0.543, 0.573 and 0.663, respectively.

Analysis of EO-AgNPs with various additions of AgNO_3_ was carried out after 24 h of initial mixing. Based on the results of the analysis, it was concluded that the greater the concentration of AgNO_3_, the higher the absorbance. AgNO_3_ concentration affects the formation of Ag nanoparticles. EO-AgNPs can be identified from UV-Vis spectra through the formation of peaks at a maximum wavelength of 430 nm. The formation of Ag nanoparticles can be seen visually from the color change and peaks that appear at a wavelength of 400–450 nm which is the maximum value of Ag nanoparticles [27, 29]. The larger precursor concentration causes the Ag reduction process to take a faster time and increase the absorbance intensity. In addition, the results of the analysis with UV-Vis spectrophotometer showed that the nanoparticles formed with AgNO_3_ concentrations of 6, 8, and 10 mM looked more stable. Qualitatively, it can be assumed that the higher the absorbance value, the more nanoparticles formed or the higher the concentration of nanoparticles in the solution.

**Figure 8:**
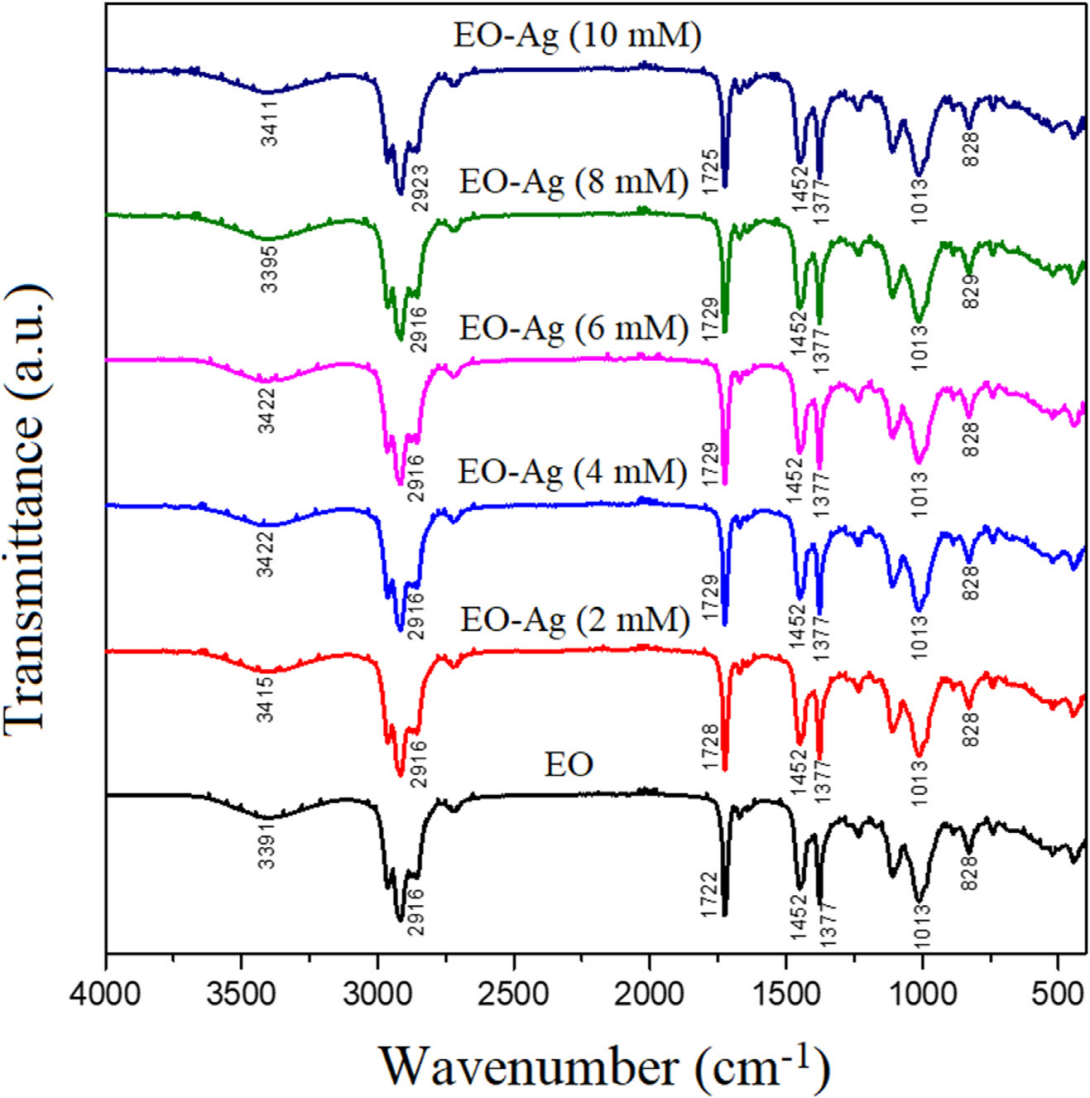
FT-IR spectra of EO-AgNPs using different concentrations of AgNO_3_.

FT-IR spectra of EO-AgNPs using different concentrations of AgNO_3_ shown in Figure 8. The wavenumbers at 3395 to 3422 cm^−1^ indicate the absorption of the OH functional group, while 2916-2923 cm^−1^, 1451 and 1377 cm^−1^, and 1013 cm^−1^ represents the absorption of Csp3-H, –CH_3,_ and CO alcohol group [47]. The FTIR spectrum also showed the presence of different functional groups. Furthermore, the absorption band of 3391cm^−1^ citronella oil is a characteristic of the stretching vibration of the O–H bond originating from the group contained in flavonoid, terpenoid, saponin, and polyphenol compounds. This is reinforced by a high absorption capacity at a wavenumber of 1013 cm^−1^ which is a stretching vibration of the C–O alcohol bond. The absorption of 1722 cm^−1^ (Figure 8) also indicates the presence of an aromatic C=C bond vibration. However, the change in the absorption intensity in the wavenumber region of Ag nanoparticles (AgNPs) did not change before being synthesized. It still showed a shift in the wavenumber indicating that there was an interaction between the OH functional group and Ag (3395-3422 cm^−1^) to form EO-AgNPs.

**Figure 9:**
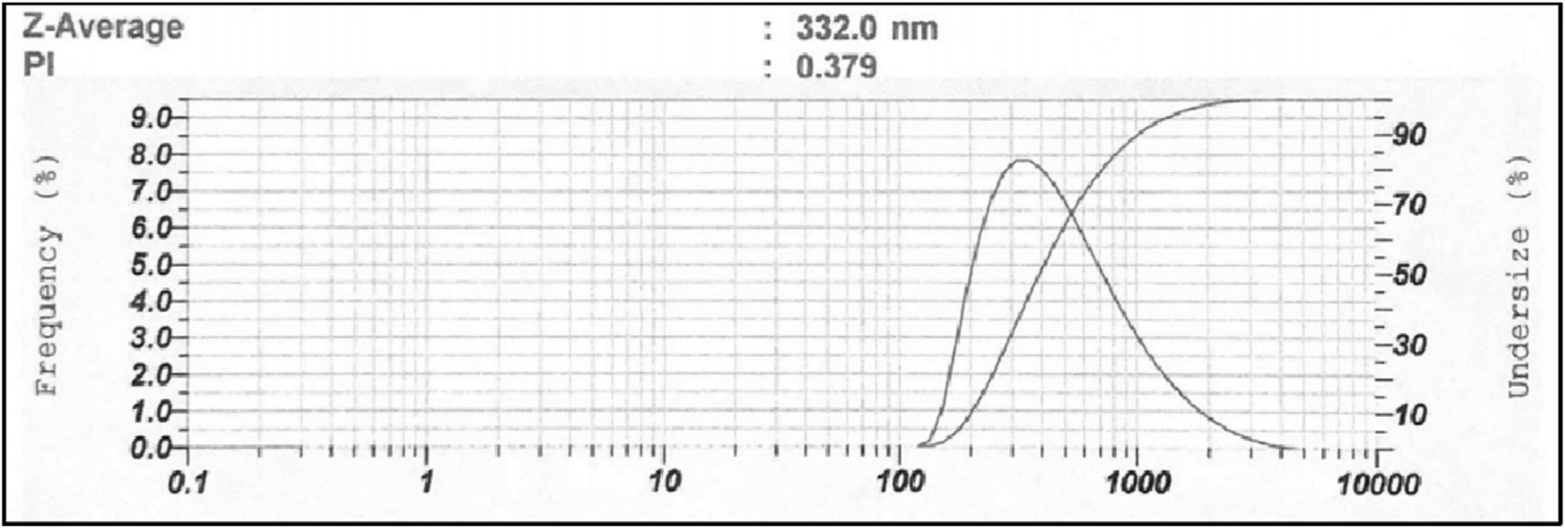
Particle size distribution EO-AgNPs.

A particle size analyzer (PSA) is a tool that uses the principle of dynamic light scattering to measure the size distribution of particles undergoing Brownian motion. Determination of particle size using PSA is faster and more accurate when compared to SEM and TEM. PSA performance is based on measurements using light. Light has a very large propagation speed so the analysis time with PSA is very short. The particle size results obtained was 332 nm and this affected the performance of the material. However, those with smaller sizes have a larger surface area which accelerate the absorption process. Increasing the amount of oil used in the formulation will increase the particle size due to a decrease in the amount of surfactant and co-surfactant used [48]. The optimal formula particle size distribution produces one peak as shown in Figure 9. This indicates that the particle size has good uniformity. Furthermore, the peak of the curve represents the distribution area of the particle size.

**Figure 10:**
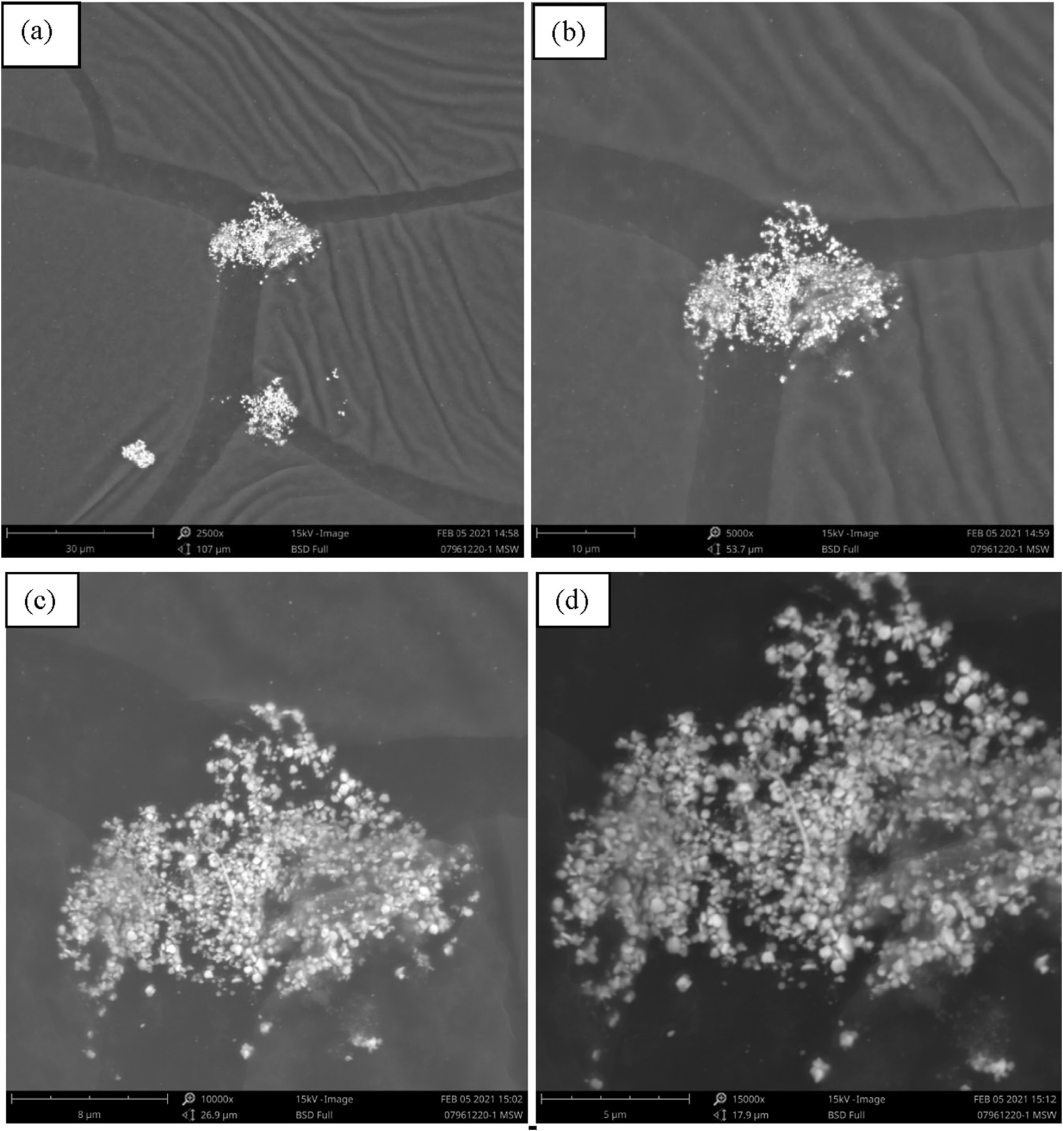
Scanning electron microscope image of EO-AgNPs with magnification (a) 2500, (b) 5000, (c) 10000 and (d) 15000x.

**Figure 11:**
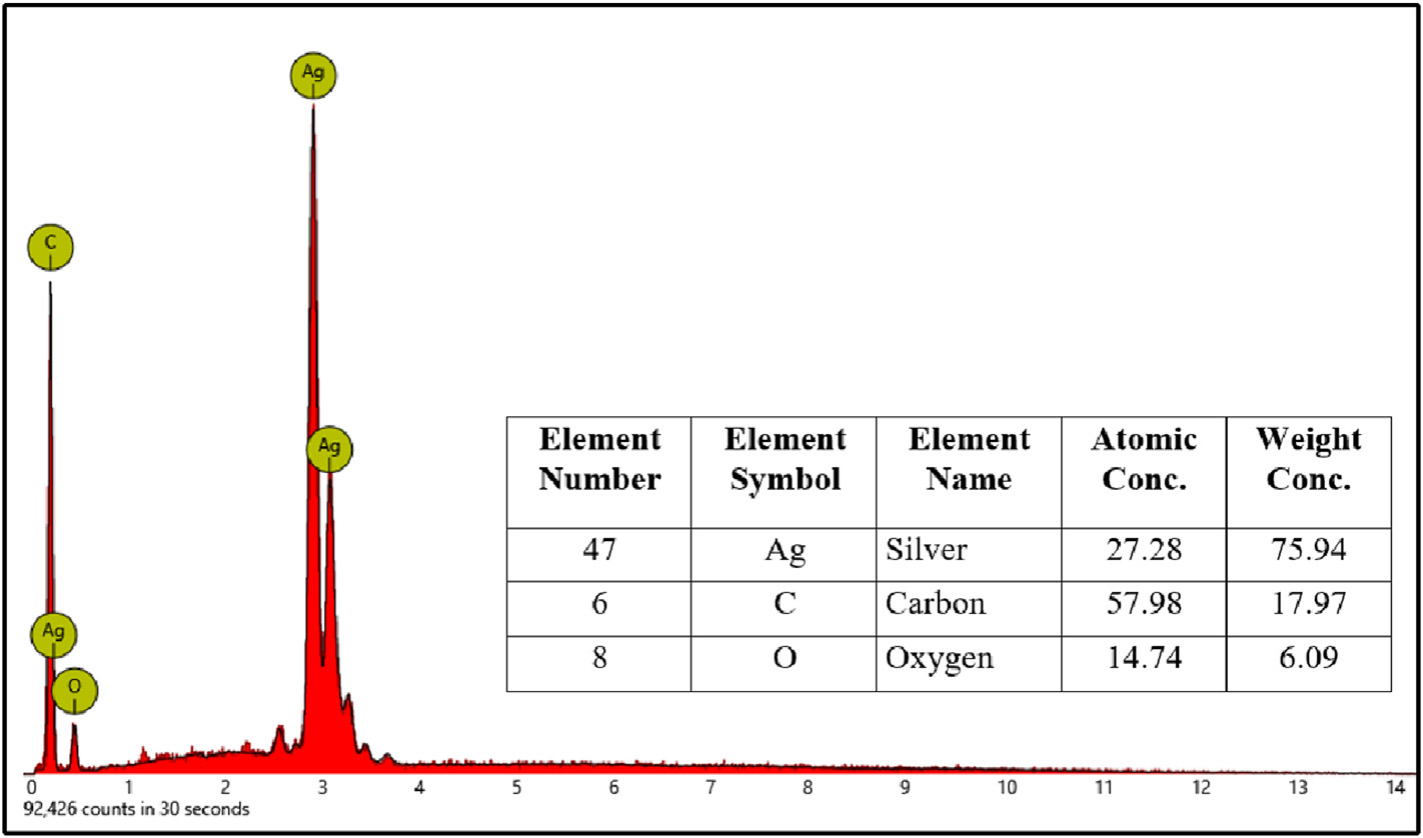
EDX spectra and elements data from EO-AgNPs.

The SEM analysis was used to view the morphology of the particles and the magnification of the Ag nanoparticle images was carried out at a scale of 2,500, 5,000, 10,000, and 15,000x. The results revealed that the particles had various round shapes and sizes which was due to the aggregation effect of nanoparticles. An example was the synthesized EO-AgNPs which had a spherical shape due to the formation of aggregates (Figure 10). The SEM data showed that the Ag nanoparticles had agglomerated. Therefore, this caused a good stability in the formation of Ag nanoparticles EO [27]. The synthesized EO-AgNPs material has been formed because lemongrass EO can act as a good bio-reductant for biosynthesis. The EDX spectra of the EO-AgNPs material are shown in Figure 11. Furthermore, the elemental composition in the EO-AgNPs consists of Ag, C, O with the percentages being 27.28, 57.98, and 14.74%, respectively.

### 3.4 Mechanism of reaction formation of the EO-AgNPs

**Figure 12:**
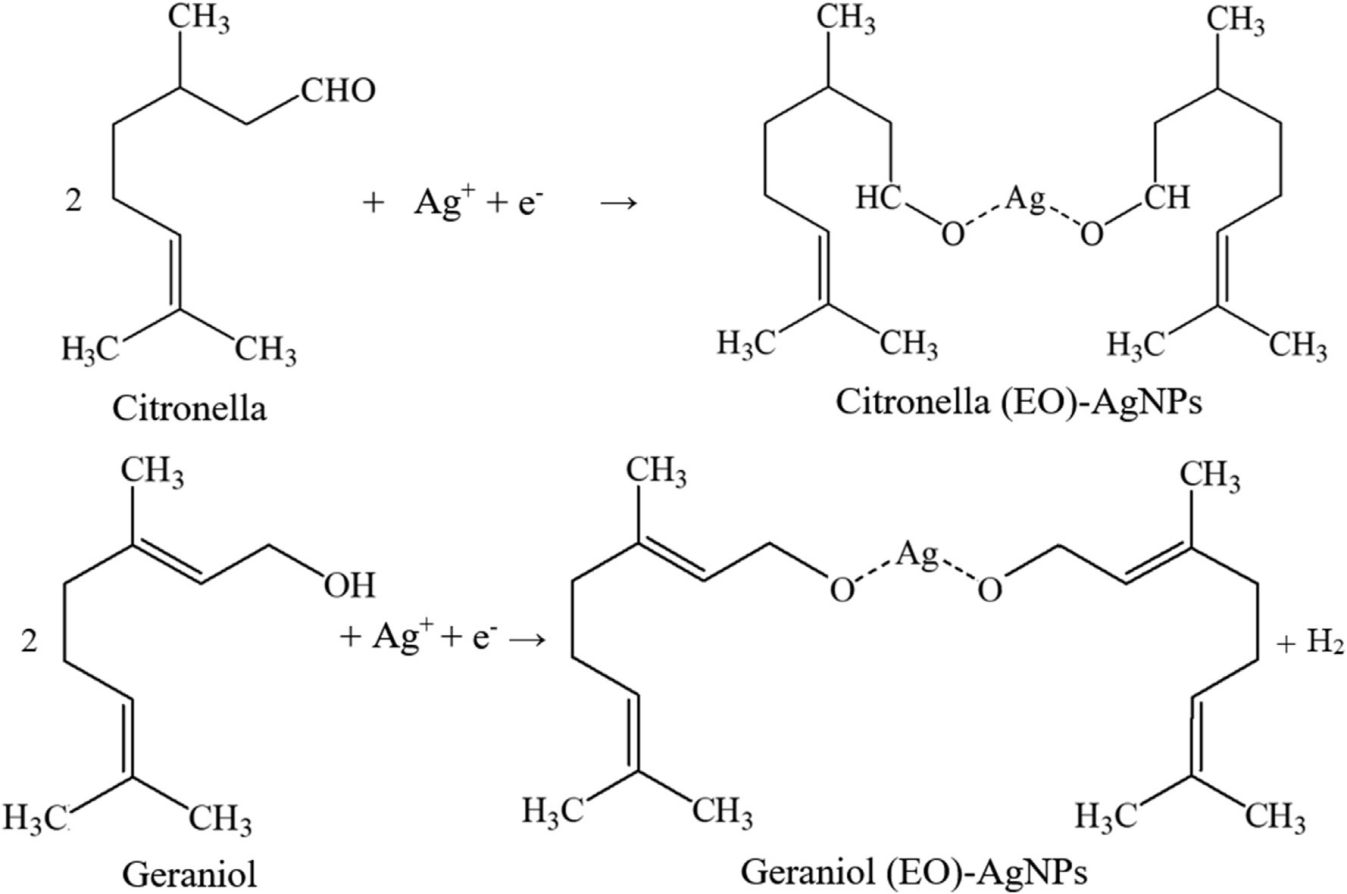
The purpose of the mechanism of reaction formation of the EO-AgNPs.

Essential oils have various types of functional groups in their molecular structure such as alcohols, ketones, aldehydes, and lactones from terpenoids, and chemical compounds such as C=C, –CH_2_, –CH_3_, -O-H, C–H, C=O, C–O–C, CH_3_–C–CH_3_, C–O, and -C-N. Figure 12 shows the purpose of the mechanism of reaction formation of the EO-AgNPs. Functional groups occupy various positions according to their ability to reduce and stabilize metals in the formation of nanoparticles, while some are involved in the synthesis of nanoparticle materials such as -O-H (Figure 12) and C=O shows its involvement [33]. Furthermore, terpenoids in EOs can be adsorbed on the metal surface of nanoparticles, possibly through interactions via pi(p)-electrons or carbonyl groups in other strong chelating agents [33]. The functional group in EO was used for Ag ion Ag (I) to Ag (0) reduction [45].

### 3.5 Applications of EO-AgNPs for inhibition of lichens on the stone surface

**Figure 13:**
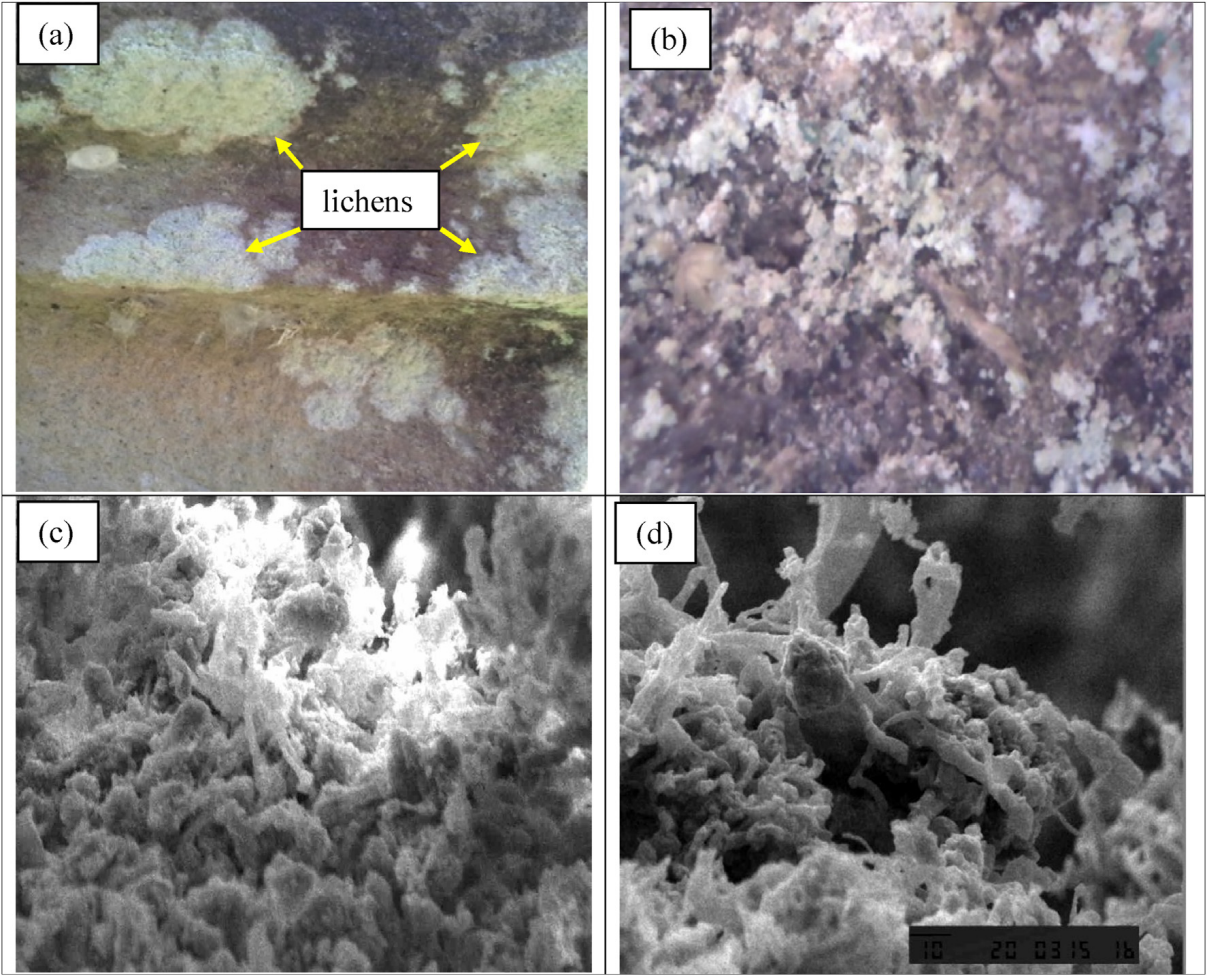
Visual of lichens on stone surface (a) lichens after treatment using EO-AgNPs (b) SEM image of lichens before treatment (c) and SEM image of lichens after treatment (d).

The Lichens are a symbiotic mutualism between algal cells and fungal mycelium that live on rocks, tree trunks, and on building walls. The types of symbiotic fungi are usually from the Ascomycota and Basidiomycota groups, while algae are usually single-celled or thread-shaped from Chlorophyta or Cyanophyta. Lichens growing on rock surfaces were green, white, and brown (Figure 13a). However, those on the rock surface after being sprayed with EO-AgNPs turned dark brown indicating that they had died (Figure 13b). Before the stone sample was analyzed using SEM, it needs to be cleaned, free of water, and then placed in a 12 mm or 25 mm sample holder. In order to attach the sample, a conductive double-sided tape is required and the sample area to be studied need to be placed at 45^o^. Afterwards, the sample was coated with Au metal with a thickness of approximately 10–30 nm, and then analyzed using SEM. Furthermore, the analysis of lichens before and after treatment with EO-AgNPs was performed using SEM (Figures 13c and 13d).

**Figure 14:**
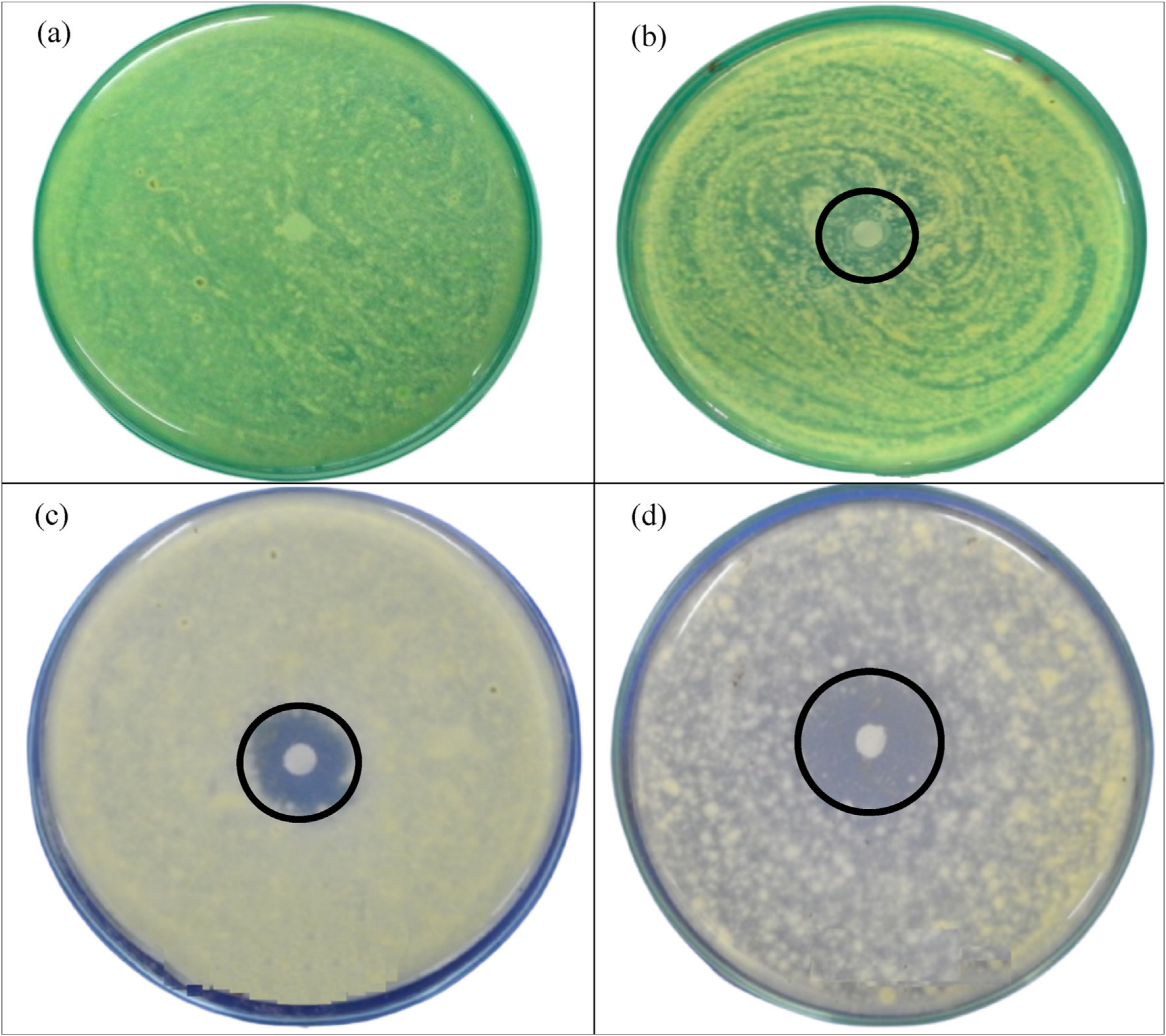
Antifungal activity for lichens using zone of inhibition of EO-AgNPs was compared against the (a) blank (distilled water) (b) control (ethanol) (c) EO and (d) EO-AgNPs.

The test results of EO-AgNPs activity against lichens using the zone of inhibition are shown in Figure 14. The EO test was carried out using the disc diffusion method which involves saturating the test material into a filter paper (paper discs). Paper discs containing certain materials were planted on a solid agar medium that had been mixed with the tested microbes, then incubated at 35 °C for 18–24 h. Furthermore, a clear zone was observed around the paper disc which indicated the absence of microbial growth. During incubation, the test material diffuses from the filter paper into the agar thereby forming an inhibition zone as shown in Figures 14c and 14d. Chemical compounds contained in EOs will diffuse into the agar medium, causing inhibition of cell wall formation. The cells are coated by a thin membrane and can rupture or be damaged. Inhibition can also occur through the process of protein synthesis. Materials such as EO-AgNPs showed a larger zone of inhibition than EO. Furthermore, EO and Ag metal both have a synergistic effect as antifungal [49]. Citronellal and geraniol are the main compounds in EO that have excellent antifungal effects [4] and are supported by Ag metal [29].

## 4 Conclusion

The synthesized EO-AgNPs material showed a brown color as a characteristic of the formation of nanoparticles. The results of the analysis using FTIR, PSA, and SEM-EDX showed that the EO-AgNPs material is a nanoparticle compound with a size of 332 nm. Furthermore, the direct testing of EO-AgNPs to kill lichens showed very effective results after 24 h of observation. The material also has a very good inhibition rate when compared to EO alone. However, the results showed that the particle size obtained was far bigger than 100 nm. The smaller the particle size, the more effective the performance of the material. Subsequently, further research needs to be carried out on the effect of stirring on the particle size of EO-AgNPs, the reaction mechanism of the active ingredients in EOs with Ag, and the application of antifungal agents.

## Declarations

### Author contribution statement

Riyanto and Nahar Cahyandaru: Conceived and designed the experiments; Analyzed and interpreted the data; Contributed reagents, materials, analysis tools or data; Wrote the paper.

Meike Mulwandari: Performed the experiments; Analyzed and interpreted the data; Contributed reagents, materials, analysis tools or data.

Luthfiah Asysyafiiyah: Performed the experiments; Analyzed and interpreted the data; Contributed reagents, materials, analysis tools or data.

Melisa I. Sirajuddin: Performed the experiments; Analyzed and interpreted the data; Contributed reagents, materials, analysis tools or data.

### Funding statement

This work was supported by the Ministry of Education, Culture, Research, and Technology of the Republic of Indonesia for financial support by research ​grants No. 311/E4.1/AK.04.PT/2021 and 3281.1/LL5/PG/2021.

### Data availability statement

Data will be made available on request.

### Declaration of interests statement

The authors declare no conflict of interest.

### Additional information

No additional information is available for this paper.
